# Supplementary material for: A Toxicity Screening Approach to Identify Bacteriophage-Encoded Anti-Microbial Proteins
Source: Viruses. 2019 Nov 14;11(11):1057. doi: 10.3390/v11111057 (PMC6893735; doi:10.3390/v11111057)

viruses

MDPI

# Supplementary information

Table S1. Primers used to amplify the non-toxic and toxic control genes.

| Primer | Sequence (restriction site underlined) | Description | Size |
|---|---|---|---|
| Non-toxic control genes amplification | | | |
| RTg121F | GCAGCGGCCGCATGAAAACCTATAATGAATTTATC | For the amplification of the ϕR1-RT g121 gene encoding the IpII internal head protein | 273 bp |
| RTg121R | GGTCCATGGTTAGGAAGCTTTTTTAAGC | | |
| RTg150F | GCAGCGGCCGCATGATTAAAGTTAATGAGC | For the amplification of the ϕR1-RT g150 gene encoding the phage DNA end protector | 1728 bp |
| RTg150R | GGTGCTAGCCTATCCAATATCAATTCGTGAA | | |
| RTg178F | GCAGCGGCCGCATGAGCAATATTAACCAGC | For the amplification of the ϕR1-RT g178 gene encoding the phage capsid vertex | 1275 bp |
| RTg178R | GGTCCATGGTTATCCTGCTATTAGTTTAGG | | |
| RTg119F | GCAGCGGCCGCATGAAAACGTATAAAGAATTTTTG | For the amplification of the ϕR1-RT g119 gene encoding the IpIII internal head protein | 591 bp |
| RTg119R | GGTCCATGGTTAACGAACGTTAGTGCCA | | |
| RTg246F | GCAGCGGCCGCATGTCTTTAAATGAAATG | For the amplification of the ϕR1-RT g246 gene encoding Phage ribonuclease H | 939 bp |
| RTg246R | GGTCCATGGTTAAAAATCATTTGCATG | | |
| Toxic control genes amplification | | | |
| NregBF | GATCGCGGCCGCCCATGACTATCAATACAGAAG | For the amplification of the T4 regB gene encoding the T4 Reg B protein | 491 bp |
| NregBR | GGCCGCTAGCTTACCTCATTGAGTTTTAATTAC | | |
| lyE174F | GCAGCGGCCGCATGGTACGCTGGACTTTGTG | For the amplification of the ϕ174 lytic gene E | 276 bp |
| lyE174R | GGTCCATGGTCACTCCTTCCGCACGTAA | | |
| NddF | GCAGCGGCCGCATGAAATATATGACTGTTACTG | For the amplification of the T4 ndd gene encoding the nuclear disruption protein (Ndd) | 456 bp |
| NddR | GGTCCATGGTTAATATGCCTGTAAAACAAA | | |

| | | | |
|---|---|---|---|
| dcd1lF | GCAGCGGCCGCAGCCTGCTGGAAAAAGGC | For the amplification of the dcd-1l gene on pETSmt3- DCD1L | 144 bp |
| dcd1lR | GGTCCATGGTTACAGCACGCTATCCAGCAC | | |
| ◎R1-RT phage holins and lysins | | | |
| RTg122F | GCAGCGGCCGCATGAATTTATTTGAAATGCTAC | For the amplification of the ϕR1-RT g122 gene encoding the lysozyme | 489 bp |
| RTg122R | GGTCCATGGTTATTTGTAGGCATCCATCG | | |
| RTg252F | GCAGCGGCCGCATGCAGACTTATAAAGAGTCTC | For the amplification of the ϕR1-RT g252 gene encoding the holin | 162 bp |
| RTg252R | GGTCCATGGTCAGCTTTTAGGTCTAAATG | | |
| RTg253F | GCAGCGGCCGCATGAGTTCGCCACGTGTTAC | For the amplification of the ϕR1-RT g253 gene encoding the holin | 657 bp |
| RTg253R | GGTCCATGGTTATCTTGATCTGCCTAACG | | |

Table S2. Primers used to amplify the HPUF genes from ϕR1-RT phage.

| Gene | Forward Primer (restriction site underlined) | Reverse Primer (restriction site underlined) | Size |
|---|---|---|---|
| g002 | GCAGCGGCCGCATGAAAATGTATGCTTATAAAAAT | GGTCCATGGTCACGGGAATACTATTTCAAC | 258 bp |
| g004 | GCAGCGGCCGCATGATTCATTCAATGAGAATTTC | GGTCCATGGTCAGGAGTTAAATCCTTCAC | 306 bp |
| g011 | GCAGCGGCCGCATGGATATTCTTACTGTGGG | GGTCCATGGTTATTCAAAAACCTTACGAAC | 378 bp |
| g012 | GCAGCGGCCGCATGAATAAGTCTGTTAATGATATCG | GGTCCATGGTTAGTCCTCAAAAATAATTCG | 393 bp |
| g014 | GCAGCGGCCGCATGTCTATTGTTAAAGTTGG | GGTCCATGGTTATAATCCAAATTCAGATTTAAG | 381 bp |
| g016 | GCAGCGGCCGCATGAAATTTCGAATTGGG | GGTCCATGGTTATTTAGAACTAAGTTCATCTTCG | 357 bp |
| g017 | GCAGCGGCCGCATGGGTGCAAAGAATATC | GGTCCATGGTTATTTAGAAGATTTAGGTAATGG | 291 bp |
| g019 | GCAGCGGCCGCATGCAAAATAATCGTGTG | GGTGCTAGCTTATTGAATAATACCATTAGGG | 270 bp |
| g022 | GCAGCGGCCGCATGCTAAAAATTGATAAACACAAT | GGTCCATGGTCATCGTAATGCCCTG | 231 bp |
| g023 | GCAGCGGCCGCATGTATAAATTGAATCCAGACC | GGTCCATGGTTAGCATAATAAGTTCTTCAATTT | 231 bp |
| g028 | GCAGCGGCCGCATGATTATGTATAAAATTAAATTTAG | GGTCCATGGTCATACAAAAGGCCACTGTG | 474 bp |
| g030 | GCAGCGGCCGCATGTATAAAGGAATCATGAGC | GGTCCATGGCTACAAAATATAATCCATAGCAATTTT | 270 bp |
| g033 | GCAGCGGCCGCATGGCTGTTGGATTTACTAAAG | GGTCCATGGTCACCACAATCTAAACAATTC | 132 bp |
| g043 | GCAGCGGCCGCATGAAACTTAAAGTTTACCGAG | GGTCCATGGTCATTTTACAAGTTCAACTTCTG | 177 bp |
| g044 | GCAGCGGCCGCATGAACTATTTAAAAATTTCAATCC | GGTCCATGGTTATTTTCTCATGCAATTATTAAATTT | 462 bp |
| g045 | GCAGCGGCCGCATGGCACGACTAATTCTTATC | GGTCCATGGTTAAGATCTAGGGGCCCAC | 561 bp |
| g049 | GCAGCGGCCGCATGTCTATTTCAACACCAGAAC | GGTCCATGGCTAGTCAGATAATTCATTAGAAGC | 198 bp |
| g061 | GCAGCGGCCGCATGAAAATCGATTCAAATCAG | GGTCCATGGTTATTTGTTAAAATAATCTTCAAGG | 201 bp |

| | | | |
|---|---|---|---|
| g064 | GCAGCGGCCGCATGAAGAAAATTGGGGTG | GGTCCATGGCTAGGGTTCCATTTGTTTTG | 276 bp |
| g065 | GCAGCGGCCGCATGCTAATTCAAACAAAATTTAAG | GGTCCATGGTTACCATAATCGTTTCCAC | 201 bp |
| g067 | GCAGCGGCCGCATGAGCCTGAAGATTAACC | GGTCCATGGTCATAATTCCTCGAAAGG | 237 bp |
| g069 | GCAGCGGCCGCATGATTAAAGTTAATTTCTTTAAACC | GGTCCATGGTTAAAATGTTTCAAGTTCTTTAAG | 369 bp |
| g070 | GCAGCGGCCGCATGAAAAATCTTGCTCGAG | GGTCCATGGTCATGATAAATCCTTAGCAG | 228 bp |
| g071 | GCAGCGGCCGCATGAAATATATTCTTTATACTAAATCC | GGTCCATGGTTATTTTATTTCCTGGATGTAAT | 207 bp |
| g073 | GCAGCGGCCGCATGATTGTATCTTCTAAAGCAAAC | GGTCCATGGTTATTTAATTTTAGAGGCAATAACC | 225 bp |
| g074 | GCAGCGGCCGCATGAAATATAAATTCAAAAGTTTTTAC | GGTCCATGGTTAGTGCCCTAATTTAAGTTTAAC | 378 bp |
| g076 | GCAGCGGCCGCGTGATTAATAAATTTTTTGCAC | GGTCCATGGTCAGATAGACGCATTTTTAG | 243 bp |
| g081 | GCAGCGGCCGCATGAAAGGCCGCG | GGTCCATGGTCATTTTTTATATTTCTCTTCTAGG | 345 bp |
| g083 | GCAGCGGCCGCATGGAAATTAAAGTCGATGAAATATA | GGTCCATGGTTAATCCTTATATTCTATATAAATGTCTG | 225 bp |
| g084 | GCAGCGGCCGCATGAAAAATCAATTAAATGAAG | GGTCCATGGTTAATTTCCATCAATAACTACC | 204 bp |
| g085 | GCAGCGGCCGCATGAATCCACTTGATATTACTTAC | GGTCCATGGTCATAAAATTACCCAGCC | 243 bp |
| g086 | GCAGCGGCCGCATGGCTCGTAAATCATTTG | GGTCCATGGTTAAGTAAAGTAAACTGATGGTTTTC | 234 bp |
| g087 | GCAGCGGCCGCATGAGTTTATTCACTGAGTTAAAATATTT | GGTCCATGGTCATTTTAGAGTTCTCAATGATTC | 879 bp |
| g089 | GCAGCGGCCGCATGTCTACATCAAAAGATATTTTAG | GGTCCATGGTTAGATAACCTTAATCTGAGAAGG | 792 bp |
| g090 | GCAGCGGCCGCATGGCTACGCATACATTTG | GGTCCATGGCTATATTTTTCCACGATTGAG | 120 bp |
| g091 | GCAGCGGCCGCATGAATAACTGGGTTGAGAATAC | GGTCCATGGTCATAAATCCTTCCATTTTAAG | 447 bp |
| g092 | GCAGCGGCCGCATGTTATATAGAATTAAATATCGTCG | GGTCCATGGTTATTCATGAGAATCATATGGAC | 438 bp |

| | | | |
|---|---|---|---|
| g093 | GCAGCGGCCGCATGGAAATCGGCG | GGTCCATGGTTACCTCAGAAATTTAGATTTAACTAC | 135 bp |
| g094 | GCAGCGGCCGCATGTCTTATATCTCTTATGTATATCAAG | GGTCCATGGTCATTTAAGTTCTAAGGATTCAC | 231 bp |
| g095 | GCAGCGGCCGCATGCGTATTAAAAAGATGTTATC | GGTCCATGGTTAAATTGTCACTGAATCATC | 216 bp |
| g096 | GCAGCGGCCGCGTGCTTATGTTAGAGTCATACAC | GGTCCATGGTCAATTTTCTAGAATAAAAGGTTC | 117 bp |
| g098 | GCAGCGGCCGCATGATTAATTTATCAAATAGTAAAAAAAA | GGTCCATGGTTAGACATTTATGTCTTTTAACG | 987 bp |
| g099 | GCAGCGGCCGCATGAAACTAGAAATAGTTGATGTG | GGTCCATGGTTAATCATAATATTTCTCCATTTTAA | 198 bp |
| g100 | GCAGCGGCCGCATGGCTATCAAATCAGTGTC | GGTCCATGGTCACCACTTAAAAGCTAACATC | 390 bp |
| g101 | GCAGCGGCCGCATGAACGAAGGTTATAATATTACATTTT | GGTCCATGGCTAGATATCAAATTTAGTAAAAGAAAAAG | 396 bp |
| g102 | GCAGCGGCCGCATGAGCATAAGCAAAGAAG | GGTCCATGGTTAATCAAAACGTACAGCG | 168 bp |
| g104 | GCAGCGGCCGCATGGAAATTGAGCTGATTAG | GGTCCATGGTCATAGATAAATATGCGTCC | 408 bp |
| g105 | GCAGCGGCCGCATGATAGGGTTCACTATACTTAAATC | GGTCCATGGTTATTCAATCTCAATAAGTGGAC | 273 bp |
| g106 | GCAGCGGCCGCATGAGTTTACTGACTTGGGC | GGTCCATGGTTATTGACCCTTATTCATAGTTG | 225 bp |
| g111 | GCAGCGGCCGCATGAGAGAACATCTTATTTCTAAAATAG | GGTCCATGGTCATTTGTCTTCCATTCC | 174 bp |
| g112 | GCAGCGGCCGCATGAAATTATTAGTGAGTATATTATTATGTG | GGTCCATGGTCATGAAATATCCTTAAATGATAAAC | 438 bp |
| g117 | GCAGCGGCCGCATGGGAACACAAGCAAC | GGTCCATGGTCAATCATTCAAATGTTCC | 204 bp |
| g124 | GCAGCGGCCGCATGAGCGTAGCAATTTTTTTG | GGTCCATGGTTAATAGTCTTCAGAACTTTCATAGTC | 264 bp |
| g126 | GCAGCGGCCGCATGTCTGTACTATTAGAAATTTCAATC | GGTCCATGGCTAGCAATCTGAACTTGATG | 213 bp |
| g127 | GCAGCGGCCGCATGGTCTATACTCCAGAACAAATAG | GGTCCATGGTTAACACATATCACTTGATGACAC | 261 bp |
| g128 | GCAGCGGCCGCATGAGTCGTTCAACTGAATTG | GGTCCATGGTTAGCAAGAAGATGATTCCC | 222 bp |

| | | | |
|---|---|---|---|
| g129 | GCAGCGGCCGCATGATATATTATTTTCCGGCC | GGTCCATGGTCATTTATTACCTCTACAATAAGTTATG | 150 bp |
| g130 | GCAGCGGCCGCATGATTTATACGACAAAAGATTATAAC | GGTCCATGGTTATACAACACGATAATCACC | 288 bp |
| g132 | GCAGCGGCCGCATGACTACTTTCAATACCGTAAC | GGTCCATGGTTAAATTAAATTGTACAACCAATTATG | 201 bp |
| g135 | GCAGCGGCCGCATGGATTTTGGTCAAAAG | GGTCCATGGTTAAGCTTTATCTTTGATAATAGG | 552 bp |
| g136 | GCAGCGGCCGCATGGGTTATATTTTAGGTTTG | GGTCCATGGTCAGAAAAAGTATATAGGTTTTACTTC | 336 bp |
| g137 | GCAGCGGCCGCATGAAAATTGCTGAACTAATTC | GGTCCATGGCTATGAGGACTTAGAAATTGTATTC | 183 bp |
| g138 | 5’GCAGCGGCCGCATGGATTATACTCATTTACAAGTG | GGTCCATGGTTATGAAACTTGGCATTTAGG | 318 bp |
| g141 | GCAGCGGCCGCATGAATACGCATCAAAAACG | GGTCCATGGTTATTTACCCGTCCAAAGAAC | 195 bp |
| g167 | GCAGCGGCCGCATGCGAAAGAATAAGAACG | GGTCCATGGCTAAAAATTGCATAAGAAGTCC | 600 bp |
| g177 | GCAGCGGCCGCATGGATATATCTATTTATAAAGATAAAAATG | GGTCCATGGTTAAATAGATTCAAATACTAAATTACCTG | 1515 bp |
| g181 | GCAGCGGCCGCATGAATAAATCAGTTGAAGTAC | GGTCCATGGTTATAAAGCAAATAACATCTGC | 234 bp |
| g199 | GCAGCGGCCGCATGGCTATTTTGCGTTTTAAG | GGTCCATGGTTAGTGACCTACAAAAGCAAG | 273 bp |
| g202 | GCAGCGGCCGCATGGATGTATTTGCAAAGC | GGTCCATGGTCAATAATTCGTGCCATAATAAG | 177 bp |
| g204 | GCAGCGGCCGCATGGTAGTTAAAATTGTAAGTATGTC | GGTCCATGGTCATTTAGTTTCTCGCTTTTTAG | 189 bp |
| g205 | GCAGCGGCCGCATGAATAAATTTATGGATGGTG | GGTCCATGGTTACTTAGATAATTCGGATTCAATG | 198 bp |
| g206 | GCAGCGGCCGCATGGACTTCTTTAAAGAAAATACC | GGTCCATGGTCAATCAGGATATTTAGTTTCAAG | 570 bp |
| g208 | GCAGCGGCCGCATGGATTTCTTCAAAGAAAAC | GGTCCATGGTTATACCTCGGTTTTGTTTAATTTATA TTG | 525 bp |
| g209 | GCAGCGGCCGCATGAACTACATTAACTTTGAACG | GGTCCATGGTTAAAAAGTAAATTCATTAGAAACTATATC | 360 bp |
| g210 | GCAGCGGCCGCATGTCCAAAAATAAAGCTAAAG | GGTCCATGGTTATACACGTGGCTTACC | 174 bp |

| | | | |
|---|---|---|---|
| g214 | GCAGCGGCCGCATGATTGATTTAATGAAACCTG | GGTCCATGGTCAACTGATAAGCTCATAGTTAATC | 315 bp |
| g217 | GCAGCGGCCGCATGATTACTAATAAAACATTTACAGAAG | GGTCCATGGTTAGTATTCCATAATTGTTTCG | 231 bp |
| g218 | GCAGCGGCCGCATGACTATTAGAACTATTGAGCTAGATG | GGTCCATGGTCATTCTAAAATCACTCTAACAAATAG | 279 bp |
| g219 | GCAGCGGCCGCATGGTAACTGTTCACGAG | GGTCCATGGTTAATGCAAAGTACGAACAG | 213 bp |
| g221 | GCAGCGGCCGCATGAAAGAGTTATTAGAAAATTATATTAAG | GGTCCATGGTTAAATCCCTAAGAAGTTTTTAAG | 288 bp |
| g222 | GCAGCGGCCGCTTGACTCAACAGGCTAATG | GGTCCATGGTCATTCTACTAAGCCTTGAAC | 132 bp |
| g224 | GCAGCGGCCGCATGAACGAATTAAACTGG | GGTCCATGGTTAAGACTTAGGAATATTAAGGTATG | 222 bp |
| g232 | GCAGCGGCCGCATGTTTGATAATATTTTTAAAAATGG | GGTCCATGGTTAATTCCTATATACCAGATTGC | 1560 bp |
| g236 | GCAGCGGCCGCATGTTCAAATTAATTCAAGAAGATG | GGTCCATGGTTAAGAATCACTTAGTTCAAATAGAATTT | 363 bp |
| g240 | GCAGCGGCCGCATGAAACGTAAAACAATTGAAC | GGTCCATGGTTAAACAAAACCTTTATCTTCG | 246 bp |
| g241 | GCAGCGGCCGCATGACCACAACTACTCAAATTG | GGTCCATGGTTATGCATCCTCTACCATATC | 243 bp |
| g255 | GCAGCGGCCGCATGAGTCATAATCTAGAACAAGTTATAG | GGTCCATGGTCACTTTGTGGGAGTATTG | 168 bp |
| g256 | GCAGCGGCCGCATGAACAATTATGTAGCTAAAAATG | GGTCCATGGTTATGACTCATAAAATTCACC | 120 bp |
| g257 | GCAGCGGCCGCATGAAAGAATTACCTTATGTAACTAAAG | GGTCCATGGTTACTGACCTTGCATTTCAC | 315 bp |
| g258 | GCAGCGGCCGCATGACTGATATTACTAAGCAGG | GGTCCATGGTTAAAGCTTCTTTTCGCG | 264 bp |
| g259 | GCAGCGGCCGCATGTGTTTGACAGTAATTGATATG | GGTCCATGGTTAGCCAATAATGATGGTTTTTTTAG | 180 bp |
| g261 | GCAGCGGCCGCATGAATTTATTAGAACGTGTAAATAAAAAA | GGTCCATGGTTATCTTGATTCACACAATTTC | 636 bp |
| g029 | GCAGCGGCCGCATGCATGCAGTAAATATTTC | GGTCCATGGTTATTTTCCTTTTGGCAC | 198 bp |
| g052 | GCAGCGGCCGCATGAAACTTTGTTTTGTTTTGATATCG | GGTCCATGGTCATAAGTTTAGTTTGTATGATTT | 366 bp |

Table S3. ϕR1-RT structural proteins identified by LC-MS/MS analysis.

| Accession | Description | Score | Coverage | Proteins | Unique Peptides | AAs | MW [kDa] |
|---|---|---|---|---|---|---|---|
| BN80_204 | Gp200 RNA polymerase-ADP-ribosyltransferase Alt 126487:128613 reverse MW:79233 | 534.13 | 80.23 | 1 | 71 | 708 | 79.2 |
| BN80_175 | Gp171 phage portal vertex of the head 104925:106502 forward MW:61051 | 236.49 | 68.95 | 1 | 48 | 525 | 61.0 |
| BN80_180 | Gp176 phage major capsid protein 108684:110261 forward MW:56829 | 377.92 | 76.95 | 1 | 46 | 525 | 56.8 |
| BN80_173 | Gp169 phage tail sheath monomer 102201:104183 forward MW:71255 | 131.65 | 53.48 | 1 | 32 | 660 | 71.2 |
| BN80_182 | Gp178 phage capsid vertex 111903:113177 forward MW:45859 | 147.19 | 67.69 | 1 | 28 | 424 | 45.8 |
| BN80_119 | Gp119 IpIII internal head protein 65450:66040 reverse MW:22411 | 117.00 | 78.06 | 1 | 25 | 196 | 22.4 |
| BN80_164 | Gp160 phage fibritin (wac) protein 94120:95565 forward MW:51789 | 123.34 | 71.73 | 1 | 23 | 481 | 51.8 |
| BN80_157 | Gp153 phage baseplate wedge 83282:85234 forward MW:72610 | 84.77 | 44.46 | 1 | 22 | 650 | 72.6 |
| BN80_251 | Gp247 phage long tail fiber proximal subunit 155079:158816 forward MW:135002 | 69.16 | 26.51 | 1 | 20 | 1245 | 134.9 |
| BN80_158 | Gp154 phage baseplate wedge initiator 85240:88338 forward MW:119162 | 41.13 | 22.29 | 1 | 16 | 1032 | 119.1 |
| BN80_121 | Gp121 IpII internal head protein 66562:66834 reverse MW:10145 | 59.92 | 67.78 | 1 | 14 | 90 | 10.1 |
| BN80_200 | Gp196 phage baseplate hub 122381:124129 forward MW:63359 | 40.02 | 31.79 | 1 | 14 | 582 | 63.3 |

| | | | | | | | |
|---|---|---|---|---|---|---|---|
| BN80_178 | Gp174 phage prohead assembly (scaffolding) protein 107185:107808 forward MW:22662 | 61.51 | 52.17 | 1 | 11 | 207 | 22.6 |
| BN80_150 | Gp146 phage DNA end protector during packaging 78168:78992 reverse MW:31043 | 30.16 | 35.40 | 1 | 11 | 274 | 31.0 |
| BN80_163 | Gp159 gp12 short tail fibers 92770:94110 forward MW:47163 | 35.86 | 34.08 | 1 | 10 | 446 | 47.1 |
| BN80_036 | Gp036 gp61.1 conserved hypothetical protein 20482:20964 reverse MW:17608 | 44.13 | 61.88 | 1 | 9 | 160 | 17.6 |
| BN80_146 | Gp142 gp57B conserved hypothetical protein 76195:76656 reverse MW:17518 | 41.39 | 65.36 | 1 | 9 | 153 | 17.5 |
| BN80_254 | Gp250 phage tail fiber-like protein 160652:162163 forward MW:54271 | 40.31 | 26.04 | 1 | 9 | 503 | 54.2 |
| BN80_192 | Gp188 DNA helicase, phage-associated 117952:118182 forward MW:8743 | 196.72 | 71.05 | 1 | 8 | 76 | 8.7 |
| BN80_246 | Gp242 single stranded DNA-binding protein, phage-associated 151853:152746 reverse MW:32464 | 28.22 | 38.05 | 1 | 8 | 297 | 32.4 |
| BN80_122 | Gp122 chain A, crystal structure of T4 lysozyme mutant T152v 66844:67332 reverse MW:18227 | 25.42 | 37.04 | 1 | 8 | 162 | 18.2 |
| BN80_161 | Gp157 phage baseplate wedge 90270:92096 forward MW:67131 | 24.25 | 13.82 | 1 | 8 | 608 | 67.1 |
| BN80_202 | Gp198 phage tail assembly 125185:126123 forward MW:34155 | 31.70 | 31.73 | 1 | 8 | 312 | 34.1 |
| BN80_154 | Gp150 T4-like phage baseplate hub + tail lysozyme 80751:82478 forward MW:63803 | 24.40 | 19.30 | 1 | 8 | 575 | 63.8 |
| P00761 | Trypsin OS=Sus scrofa PE=1 SV=1 - [TRYP_PIG] | 65.12 | 25.11 | 1 | 6 | 231 | 24.4 |
| BN80_153 | Gp149 phage baseplate wedge 80176:80754 forward MW:22194 | 31.01 | 37.50 | 1 | 6 | 192 | 22.2 |

| | | | | | | | |
|---|---|---|---|---|---|---|---|
| BN80_198 | Gp194 phage baseplate hub subunit 120749:121888 forward MW:43058 | 16.87 | 24.27 | 1 | 6 | 379 | 43.0 |
| BN80_159 | Gp155 phage baseplate wedge subunit (T4-like gp8) 88331:89335 forward MW:38139 | 14.43 | 23.05 | 1 | 6 | 334 | 38.1 |
| BN80_253 | Gp249 tail fiber protein p36 (protein Gp36) 159972:160619 forward MW:23070 | 19.18 | 40.93 | 1 | 6 | 215 | 23.1 |
| BN80_190 | Gp186 Inh inhibitor of prohead protease gp21 115594:116301 reverse MW:26921 | 19.86 | 29.79 | 1 | 5 | 235 | 26.9 |
| BN80_179 | Gp175 phage prohead assembly (scaffolding) protein 107841:108665 forward MW:30458 | 16.32 | 28.83 | 1 | 5 | 274 | 30.4 |
| BN80_165 | Gp161 T4-like phage head completion, neck hetero-dimeric protein (T4-like gp13) 95624:96556 forward MW:34703 | 9.54 | 15.48 | 1 | 5 | 310 | 34.7 |
| BN80_006 | Gp006 rIIB protector from prophage-induced early lysis 2297:3172 reverse MW:32780 | 21.58 | 21.31 | 1 | 4 | 291 | 32.8 |
| BN80_166 | Gp162 T4-like phage head completion, neck hetero-dimeric protein (T4-like gp14) 96560:97366 forward MW:30661 | 23.14 | 24.25 | 1 | 4 | 268 | 30.6 |
| BN80_201 | Gp197 phage baseplate tail tube cap (T4-like gp48) 124139:125185 forward MW:37991 | 15.13 | 16.09 | 1 | 4 | 348 | 38.0 |
| BN80_239 | Gp235 thymidylate synthase 148344:149204 reverse MW:32716 | 17.55 | 17.13 | 1 | 4 | 286 | 32.7 |
| BN80_216 | Gp212 phage head assembly chaperone protein 134388:134711 reverse MW:11631 | 12.45 | 48.60 | 1 | 4 | 107 | 11.6 |
| BN80_252 | Gp248 tail fiber protein Gp35 158816:159946 forward MW:41604 | 15.01 | 19.41 | 1 | 4 | 376 | 41.6 |
| BN80_160 | Gp156 phage baseplate wedge 89404:90270 forward MW:31166 | 13.74 | 21.18 | 1 | 3 | 288 | 31.1 |

| | | | | | | | |
|---|---|---|---|---|---|---|---|
| BN80_177 | Gp173 phage capsid and scaffold 106763:107185 forward MW:15923 | 7.54 | 8.57 | 1 | 3 | 140 | 15.9 |
| BN80_055 | Gp055 DNA polymerase, phage-associated 32894:35599 reverse MW:103949 | 9.71 | 4.44 | 1 | 3 | 901 | 103.9 |
| BN80_003 | Gp003 DenB DNA endonuclease IV 993:1550 reverse MW:20475 | 12.83 | 18.38 | 1 | 3 | 185 | 20.5 |
| BN80_237 | Gp233 ribonucleotide reductase of class Ia (aerobic),alpha subunit 145317:147566 reverse MW:84645 | 8.96 | 7.48 | 1 | 3 | 749 | 84.6 |
| BN80_108 | Gp108 rI.1 conserved hypothetical protein 60576:60788 reverse MW:8224 | 7.90 | 48.57 | 1 | 3 | 70 | 8.2 |
| BN80_131 | Gp131 Phage protein 70869:71468 reverse MW:22372 | 10.89 | 24.62 | 1 | 3 | 199 | 22.4 |
| BN80_031 | Gp031 dCTP pyrophosphatase, phage-associated 17378:17896 reverse MW:20099 | 6.92 | 27.91 | 1 | 3 | 172 | 20.1 |
| BN80_082 | Gp082 thioredoxin, phage-associated 48532:48804 reverse MW:10397 | 6.18 | 47.78 | 1 | 3 | 90 | 10.4 |
| BN80_024 | Gp024 DNA helicase, phage-associated 13006:14325 reverse MW:49468 | 3.36 | 10.93 | 1 | 3 | 439 | 49.4 |
| BN80_042 | Gp042 Phage recombination protein 23615:24793 reverse MW:43475 | 2.03 | 11.48 | 1 | 2 | 392 | 43.4 |
| BN80_168 | Gp164 proximal tail sheath stabilization protein 98021:98842 forward MW:31648 | 18.30 | 14.65 | 1 | 2 | 273 | 31.6 |
| BN80_135 | Gp134 hypothetical protein 72420:72578 reverse MW:5824 | 7.14 | 21.15 | 1 | 2 | 52 | 5.8 |
| BN80_211 | Gp207 hypothetical protein 132444:132713 reverse MW:9848 | 8.45 | 34.83 | 1 | 2 | 89 | 9.8 |
| BN80_025 | Gp025 hypothetical protein 14322:14603 reverse MW:11206 | 7.27 | 29.03 | 1 | 2 | 93 | 11.2 |

| | | | | | | | |
|---|---|---|---|---|---|---|---|
| BN80_008 | Gp008 hypothetical protein 5456:5608 reverse MW:5952 | 1.87 | 18.00 | 1 | 2 | 50 | 5.9 |
| BN80_227 | Gp223 hypothetical protein 138442:138948 reverse MW:19289 | 0.00 | 22.02 | 1 | 2 | 168 | 19.3 |
| BN80_224 | Gp220 3'-phosphatase, 5'-polynucleotide kinase, phage-associated 137074:137985 reverse MW:34976 | 2.66 | 7.92 | 1 | 2 | 303 | 35.0 |
| BN80_046 | Gp046 RB69ORF047c hypothetical protein 26119:27033 reverse MW:35349 | 2.71 | 9.54 | 1 | 2 | 304 | 35.3 |
| BN80_162 | Gp158 hypothetical protein 92096:92770 forward MW:23844 | 3.17 | 12.95 | 1 | 2 | 224 | 23.8 |
| BN80_205 | Gp201 DNA ligase, phage-associated 128712:130205 reverse MW:56745 | 5.40 | 4.83 | 1 | 2 | 497 | 56.7 |
| BN80_050 | Gp050 RB69ORF050c hypothetical protein 28231:29358 reverse MW:43593 | 2.03 | 6.67 | 1 | 2 | 375 | 43.6 |
| BN80_077 | Gp077 hypothetical protein 46018:46182 reverse MW:6178 | 4.65 | 25.93 | 1 | 1 | 54 | 6.2 |
| BN80_133 | Gp133 hypothetical protein 72080:72316 reverse MW:8844 | 7.11 | 20.51 | 1 | 1 | 78 | 8.8 |
| BN80_080 | Gp080 gp49.2 hypothetical protein 47873:48181 reverse MW:12202 | 2.80 | 19.61 | 1 | 1 | 102 | 12.2 |
| BN80_048 | Gp048 hypothetical protein 27721:28014 reverse MW:11383 | 2.60 | 17.53 | 1 | 1 | 97 | 11.4 |
| BN80_176 | Gp172 Gp 67 prohead core protein, precursor to internal peptides 106503:106769 forward MW:10039 | 3.44 | 17.05 | 1 | 1 | 88 | 10.0 |
| BN80_068 | Gp068 T4-like phage RNA polymerase sigma factor for late transcription 42770:43117 reverse MW:13321 | 2.72 | 16.52 | 1 | 1 | 115 | 13.3 |
| BN80_215 | Gp211 rIII lysis inhibition accessory protein, rapid lysis phenotype 134055:134303 reverse MW:9305 | 3.12 | 15.85 | 1 | 1 | 82 | 9.3 |

| | | | | | | | |
|---|---|---|---|---|---|---|---|
| BN80_013 | Gp013 hypothetical protein 8527:8967 reverse MW:17210 | 0.00 | 14.38 | 1 | 1 | 146 | 17.2 |
| BN80_232 | Gp228 Alc inhibitor of host transcription 140371:140877 reverse MW:19158 | 0.00 | 14.29 | 1 | 1 | 168 | 19.1 |
| BN80_037 | Gp037 hypothetical protein 20966:21157 reverse MW:7409 | 0.00 | 14.29 | 1 | 1 | 63 | 7.4 |
| BN80_005 | Gp005 hypothetical protein 2047:2262 reverse MW:8389 | 2.28 | 14.08 | 1 | 1 | 71 | 8.4 |
| BN80_060 | Gp060 RpbA RNA polymerase binding protein, function unknown 38375:38788 reverse MW:15518 | 3.74 | 12.41 | 1 | 1 | 137 | 15.5 |
| BN80_149 | Gp145 phage tail completion protein 77580:78158 reverse MW:21385 | 4.67 | 11.46 | 1 | 1 | 192 | 21.4 |
| BN80_079 | Gp079 hypothetical protein 47420:47863 reverse MW:16881 | 2.66 | 7.48 | 1 | 1 | 147 | 16.9 |
| BN80_018 | Gp018 hypothetical protein 10297:10788 reverse MW:18051 | 1.71 | 6.75 | 1 | 1 | 163 | 18.0 |
| BN80_110 | Gp110 Phage protein 61596:62051 reverse MW:16527 | 0.00 | 6.62 | 1 | 1 | 151 | 16.5 |
| BN80_220 | Gp216 hypothetical protein 135943:136314 reverse MW:13690 | 2.23 | 6.50 | 1 | 1 | 123 | 13.7 |
| BN80_229 | Gp225 hypothetical protein 139178:139654 reverse MW:18355 | 0.00 | 6.33 | 1 | 1 | 158 | 18.3 |
| BN80_172 | Gp168 phage terminase, large subunit 100784:102166 forward MW:52254 | 0.00 | 5.65 | 1 | 1 | 460 | 52.2 |

Table S4. Hypothetical proteins annotated as structural proteins from the LC-MS/MS analysis.

| Accession | Description | Score | Coverage | # Proteins | # Unique Peptides | # AAs | MW [kDa] |
|---|---|---|---|---|---|---|---|

| | | | | | | | |
|---|---|---|---|---|---|---|---|
| BN80_ 036 | Gp036 Gp61.1 conserved hypothetical protein 20482:20964 reverse MW:17608 | 44.1 3 | 61.88 | 1 | 9 | 160 | 17.6 |
| BN80_ 146 | Gp142 Gp57B conserved hypothetical protein 76195:76656 reverse MW:17518 | 41.3 9 | 65.36 | 1 | 9 | 153 | 17.5 |
| BN80_ 108 | Gp108 rI.1 conserved hypothetical protein 60576:60788 reverse MW:8224 | 7.90 | 48.57 | 1 | 3 | 70 | 8.2 |
| BN80_ 135 | Gp134 hypothetical protein 72420:72578 reverse MW:5824 | 7.14 | 21.15 | 1 | 2 | 52 | 5.8 |
| BN80_ 211 | Gp207 hypothetical protein 132444:132713 reverse MW:9848 | 8.45 | 34.83 | 1 | 2 | 89 | 9.8 |
| BN80_ 025 | Gp025 hypothetical protein 14322:14603 reverse MW:11206 | 7.27 | 29.03 | 1 | 2 | 93 | 11.2 |
| BN80_ 008 | Gp008 hypothetical protein 5456:5608 reverse MW:5952 | 1.87 | 18.00 | 1 | 2 | 50 | 5.9 |
| BN80_ 227 | Gp223 hypothetical protein 138442:138948 reverse MW:19289 | 0.00 | 22.02 | 1 | 2 | 168 | 19.3 |
| BN80_ 162 | Gp158 hypothetical protein 92096:92770 forward MW:23844 | 3.17 | 12.95 | 1 | 2 | 224 | 23.8 |
| BN80_ 050 | Gp050 RB69ORF050c hypothetical protein 28231:29358 reverse MW:43593 | 2.03 | 6.67 | 1 | 2 | 375 | 43.6 |
| BN80_ 077 | Gp077 hypothetical protein 46018:46182 reverse MW:6178 | 4.65 | 25.93 | 1 | 1 | 54 | 6.2 |
| BN80_ 133 | Gp133 hypothetical protein 72080:72316 reverse MW:8844 | 7.11 | 20.51 | 1 | 1 | 78 | 8.8 |
| BN80_ 080 | Gp080 Gp 49.2 hypothetical protein 47873:48181 reverse MW:12202 | 2.80 | 19.61 | 1 | 1 | 102 | 12.2 |
| BN80_ 048 | Gp048 hypothetical protein 27721:28014 reverse MW:11383 | 2.60 | 17.53 | 1 | 1 | 97 | 11.4 |

| | | | | | | | |
|---|---|---|---|---|---|---|---|
| BN80_ 013 | Gp013 hypothetical protein 8527:8967 reverse MW:17210 | 0.00 | 14.38 | 1 | 1 | 146 | 17.2 |
| BN80_ 037 | Gp037 hypothetical protein 20966:21157 reverse MW:7409 | 0.00 | 14.29 | 1 | 1 | 63 | 7.4 |
| BN80_ 005 | Gp005 hypothetical protein 2047:2262 reverse MW:8389 | 2.28 | 14.08 | 1 | 1 | 71 | 8.4 |
| BN80_ 079 | Gp079 hypothetical protein 47420:47863 reverse MW:16881 | 2.66 | 7.48 | 1 | 1 | 147 | 16.9 |
| BN80_ 018 | Gp018 hypothetical protein 10297:10788 reverse MW:18051 | 1.71 | 6.75 | 1 | 1 | 163 | 18.0 |
| BN80_ 220 | Gp216 hypothetical protein 135943:136314 reverse MW:13690 | 2.23 | 6.50 | 1 | 1 | 123 | 13.7 |
| BN80_ 229 | Gp225 hypothetical protein 139178:139654 reverse MW:18355 | 0.00 | 6.33 | 1 | 1 | 158 | 18.3 |

Table S5. BLASTp and HHpred analysis results for the HPUFs. The results are based on the BLASTp and HHpred analysis run online during the month of January 2018.

| No | HPUF | Protein ID | BLASTP | | HHpred |
|---|---|---|---|---|---|
| | | | Major Hit | Putative conserved domains | Major Hit |
| 1 | Gp002 | CCI88576.1 | Hypothetical phage protein | Not detected | No significant hits |
| 2 | Gp004 | CCI88578.1 | Hypothetical phage protein | Not detected | No significant hits |
| 3 | Gp011 | CCI88585.1 | Hypothetical phage protein | Not detected | No significant hits |
| 4 | Gp012 | CCI88586.1 | Hypothetical phage protein | ribonuclease Z | No significant hits |
| 5 | Gp014 | CCI88588.1 | Hypothetical phage protein | Not detected | No significant hits |
| 6 | Gp016 | CCI88590.1 | Hypothetical phage protein | Not detected | No significant hits |
| 7 | Gp017 | CCI88591.1 | Hypothetical phage protein, DUF551 domain-containing protein | Not detected | Protein of unknown function (DUF551) |

| | | | | | |
|---|---|---|---|---|---|
| 8 | Gp019 | CCI88593.1 | No significant hits | Not detected | No significant hits |
| 9 | Gp022 | CCI88596.1 | Hypothetical phage protein | Not detected | No significant hits |
| 10 | Gp023 | CCI88597.1 | Hypothetical phage protein | Not detected | No significant hits |
| 11 | Gp028 | CCI88602.1 | Hypothetical phage protein | Not detected | No significant hits |
| 12 | Gp029 | CCI88603.1 | Hypothetical phage protein | The SMC (structural maintenance of chromosomes) superfamily proteins | No significant hits |
| 13 | Gp030 | CCI88604.1 | Hypothetical phage protein | Not detected | No significant hits |
| 14 | Gp032 | CCI88606.1 | Hypothetical phage protein | Not detected | Nucleoside 2-deoxyribosyltransferase like |
| 15 | Gp033 | CCI88607.1 | Hypothetical phage protein | Prokaryotic dksA/traR C4-type zinc finger | Zn finger |
| 16 | Gp038 | CCI88612.1 | Hypothetical phage proteins, Spackle periplasmic protein from Enterobacter phage PG7 | Not detected | Adhesin |
| 17 | Gp043 | CCI88617.1 | Hypothetical phage protein | Not detected | No significant hits |
| 18 | Gp044 | CCI88618.1 | Hypothetical phage protein | Uncharacterized protein, contains a C-terminal ATPase domain (Function unknown) | No significant hits |
| 19 | Gp045 | CCI88619.1 | Hypothetical phage protein | Not detected | Thymidylate kinase |

| | | | | | |
|---|---|---|---|---|---|
| 20 | Gp047 | CCI88621.1 | Hypothetical phage protein, dCMP hydroxymethylase in phage | Thymidylate synthase and pyrimidine hydroxymethylase | CMP 5-hydroxymethylase |
| 21 | Gp049 | CCI88623.1 | Hypothetical phage protein | Not detected | No significant hits |
| 22 | Gp052 | CCI88626.1 | capsule biosynthesis protein of Serratia phage CHI14 | Haloacid Dehalogenase-like Hydrolases | Polynucleotide kinase |
| 23 | Gp053 | CCI88627.1 | bifunctional protein GlmU of E. coli O157 typing phage 3 | GCD1 superfamily= NDP-sugar pyrophosphorylase, includes eIF-2Bgamma, eIF-2Bepsilon, and LPS biosynthesis | MurU; Nucleotidyltransferase family protein, uridyltransferase |
| 24 | Gp061 | CCI88635.1 | Hypothetical phage protein | Not detected | Phage protein Gp45.2 |
| 25 | Gp064 | CCI88638.1 | Hypothetical phage protein | Not detected | Non heme iron peroxidases |
| 26 | Gp065 | CCI88639.1 | Hypothetical phage protein | Not detected | No significant hits |
| 27 | Gp067 | CCI88641.1 | Hypothetical phage protein | Not detected | Family of unknown function (DUF5491) |
| 28 | Gp069 | CCI88643.1 | Hypothetical phage protein | Not detected | No significant hits |
| 29 | Gp070 | CCI88644.1 | Hypothetical phage protein | Not detected | Family of unknown function (DUF5495) - This is a family of unknown function found in Myoviridae. |

| | | | | | |
|---|---|---|---|---|---|
| 30 | Gp071 | CCI88645.1 | Hypothetical phage protein | Not detected | No significant hits |
| 31 | Gp073 | CCI88647.1 | Hypothetical phage protein | Not detected | No significant hits |
| 32 | Gp074 | CCI88648.1 | Hypothetical phage protein | Not detected | No significant hits |
| 33 | Gp075 | CCI88649.1 | Hypothetical phage protein, glutaredoxin in Pectobacterium bacteriophage PM2 | Not detected | No significant hits |
| 34 | Gp076 | CCI88650.1 | Hypothetical phage protein | Seven-transmembrane G protein-coupled receptor superfamily | Detected 1 transmembrane helix |
| 35 | Gp081 | CCI88655.1 | Hypothetical phage protein | Not detected | No significant hits |
| 36 | Gp083 | CCI88657.1 | Hypothetical phage protein | Not detected | Translation initiation factor 2 beta |
| 37 | Gp084 | CCI88658.1 | Hypothetical phage protein, thioredoxin in Escherichia phage EC121 | Not detected | Transposase zinc-ribbon domain |
| 38 | Gp085 | CCI88659.1 | Hypothetical phage protein | Not detected | No significant hits |
| 39 | Gp086 | CCI88660.1 | Hypothetical phage protein | Not detected | No significant hits |
| 40 | Gp087 | CCI88661.1 | Hypothetical phage protein | Not detected | Detected coiled-coil segments |
| 41 | Gp089 | CCI88663.1 | Hypothetical phage protein | Not detected | No significant hits |
| 42 | Gp090 | CCI88664.1 | Hypothetical phage protein | Not detected | No significant hits |
| 43 | Gp091 | CCI88665.1 | Hypothetical phage protein | Not detected | No significant hits |
| 44 | Gp092 | CCI88666.1 | Hypothetical phage protein | Not detected | No significant hits |
| 45 | Gp093 | CCI88667.1 | Hypothetical phage protein | Not detected | No significant hits |
| 46 | Gp094 | CCI88668.1 | Hypothetical phage protein | Not detected | Detected 1 transmembrane helix |
| 47 | Gp095 | CCI88669.1 | Hypothetical phage protein | Not detected | DNA-directed RNA polymerase II subunit |

| | | | | | |
|---|---|---|---|---|---|
| 48 | Gp096 | CCI88670.1 | Hypothetical phage protein | Not detected | No significant hits |
| 49 | Gp098 | CCI88672.1 | Hypothetical phage protein | Not detected | Detected coiled-coil segments |
| 50 | Gp099 | CCI88673.1 | Hypothetical phage protein | Not detected | No significant hits |
| 51 | Gp100 | CCI88674.1 | Hypothetical phage protein | Not detected | No significant hits |
| 52 | Gp101 | CCI88675.1 | Hypothetical phage protein | Not detected | No significant hits |
| 53 | Gp102 | CCI88676.1 | Hypothetical phage protein | Not detected | No significant hits |
| 54 | Gp104 | CCI88678.1 | Hypothetical phage protein | Not detected | No significant hits |
| 55 | Gp105 | CCI88679.1 | Hypothetical phage protein | Not detected | Detected 2 transmembrane helices |
| 56 | Gp106 | CCI88680.1 | Hypothetical phage protein | Not detected | Uncharacterized protein |
| 57 | Gp107 | CCI88681.1 | rI lysis inhibition regulator membrane protein of Yersinia phage vB_YenM_TG1 | hypothetical protein | No significant hits |
| 58 | Gp111 | CCI88685.1 | Hypothetical phage protein | Not detected | No significant hits |
| 59 | Gp112 | CCI88686.1 | Hypothetical phage protein | Not detected | No significant hits |
| 60 | Gp117 | CCI88691.1 | Hypothetical phage protein | Not detected | No significant hits |
| 61 | Gp124 | CCI88698.1 | Hypothetical phage protein | Not detected | No significant hits |
| 62 | Gp126 | CCI88700.1 | Hypothetical phage protein | Not detected | No significant hits |
| 63 | Gp127 | CCI88701.1 | Hypothetical phage protein | Sigma-70 region 3 ; Region 3 forms a discrete compact three helical domain within the sigma-factor. | No significant hits |
| 64 | Gp128 | CCI88702.1 | Hypothetical phage protein | cl10376, hypothetical protein | No significant hits |

| | | | | | |
|---|---|---|---|---|---|
| 65 | Gp129 | CCI88703.1 | Hypothetical phage protein | Not detected | No significant hits |
| 66 | Gp130 | CCI88704.1 | Hypothetical phage protein | Not detected | No significant hits |
| 67 | Gp132 | CCI88706.1 | Hypothetical phage protein | Not detected | No significant hits |
| 68 | Gp135 | CCI88709.1 | Hypothetical phage protein | Not detected | No significant hits |
| 69 | Gp136 | CCI88710.1 | Hypothetical phage protein | Not detected | No significant hits |
| 70 | Gp137 | CCI88711.1 | Hypothetical phage protein | Not detected | Detected 2 transmembrane helices |
| 71 | Gp138 | CCI88712.1 | No significant hits | Not detected | No significant hits |
| 72 | Gp139 | CCI88713.1 | Hypothetical phage protein | YadA-like C-terminal region, | Adhesin yadA |
| 73 | Gp141 | CCI88715.1 | Hypothetical phage protein | Not detected | No significant hits |
| 74 | Gp143 | CCI88717.1 | Hypothetical protein Yersinia phage fHe-Yen9-01, Yersinia phage vB_YenM_TG1 | Not detected | Detected coiled-coil segments, GP57- Phage Tail fiber assembly helper protein |
| 75 | Gp167 | CCI88741.1 | Hypothetical phage protein | TnsA endonuclease N terminal | endodeoxyribonuclease I |
| 76 | Gp177 | CCI88751.1 | Hypothetical protein Yersinia phage fHe-Yen9-01, Yersinia phage vB_YenM_TG1 | putative Hef-like homing endonuclease | Very short patch repair (VSR) endonuclease |
| 77 | Gp179 | CCI88753.1 | RNA ligase of Yersinia phage vB_YenM_TG1 | Adenylation domain of proteins similar to ATP-dependent polynucleotide ligases | LYSINE ADENYLATE |

| | | | | | |
|---|---|---|---|---|---|
| 78 | Gp181 | CCI88755.1 | Hypothetical phage protein | Protein of unknown function (DUF2774) ;This is a viral family of proteins with unknown function | |
| 79 | Gp183 | CCI88757.1 | Hypothetical phage protein | Not detected | Capsid vertex protein gp24 |
| 80 | Gp184 | CCI88758.1 | Head outer phage capsid protein | Not detected | Capsid vertex protein gp24 |
| 81 | Gp185 | CCI88759.1 | Head outer phage capsid protein | Not detected | Capsid vertex protein gp24 |
| 82 | Gp199 | CCI88773.1 | Hypothetical phage protein | Not detected | No significant hits |
| 83 | Gp202 | CCI88776.1 | Hypothetical phage protein | Not detected | No significant hits |
| 84 | Gp204 | CCI88778.1 | Hypothetical phage protein | Not detected | No significant hits |
| 85 | Gp205 | CCI88779.1 | Hypothetical phage protein | Not detected | Protein of unknown function (DUF2945) |
| 86 | Gp206 | CCI88780.1 | Hypothetical phage protein | Not detected | No significant hits |
| 87 | Gp208 | CCI88782.1 | Hypothetical phage protein | Not detected | No significant hits |
| 88 | Gp209 | CCI88783.1 | Hypothetical phage protein | Phage Gp30.7 protein of unknown function | Phage Gp30.7 protein. Family members seem to be exclusively from the T4-like viruses. The function of this family is unknown. |
| 89 | Gp210 | CCI88784.1 | Hypothetical phage protein | Not detected | No significant hits |

| | | | | | |
|---|---|---|---|---|---|
| 90 | Gp213 | CCI88787.1 | Tail fiber protein of Enterobacteria phage RB3 and Shigella phage SHFML-11 | Protein of unknown function (DUF2693) | WYL_2, Sm-like SH3 beta-barrel fold |
| 91 | Gp214 | CCI88788.1 | Hypothetical phage protein | Not detected | No significant hits |
| 92 | Gp217 | CCI88791.1 | Hypothetical phage protein | Not detected | DinI; DinI-like family |
| 93 | Gp218 | CCI88792.1 | Hypothetical phage protein | Not detected | no significant hits |
| 94 | Gp219 | CCI88793.1 | Hypothetical phage protein | Not detected | no significant hits |
| 95 | Gp221 | CCI88795.1 | Hypothetical phage protein | Not detected | No significant hits |
| 96 | Gp222 | CCI88796.1 | Hypothetical phage protein | Not detected | no significant hits |
| 97 | Gp224 | CCI88798.1 | Hypothetical phage protein | Protein of uncharacterised function (DUF551) | DUF551; Protein of unknown function (DUF551) |
| 98 | Gp232 | CCI88806.1 | Putative Hef-like homing endonuclease of Salmonella phage SG1 | Not detected | No significant hits |
| 99 | Gp236 | CCI88810.1 | Hypothetical phage protein | Not detected | No significant hits |
| 100 | Gp240 | CCI88814.1 | Hypothetical phage protein | Not detected | No significant hits |
| 101 | Gp241 | CCI88815.1 | Hypothetical phage protein | Not detected | Bacteriophage FRD3 protein |
| 102 | Gp251 | CCI88825.1 | Hypothetical phage protein | Caudovirales tail fibre assembly protein | Caudovirales tail fibre assembly protein |
| 103 | Gp255 | CCI88829.1 | No significant hits | Not detected | No significant hits |
| 104 | Gp256 | CCI88830.1 | Hypothetical phage protein | Not detected | No significant hits |
| 105 | Gp257 | CCI88831.1 | Hypothetical phage protein | Not detected | No significant hits |
| 106 | Gp258 | CCI88832.1 | Hypothetical phage protein | Not detected | No significant hits |
| 107 | Gp259 | CCI88833.1 | Hypothetical phage protein | Not detected | No significant hits |

| | | | | | |
|---|---|---|---|---|---|
| 108 | Gp261 | CCI88835.1 | Hypothetical phage protein, sprT domain-containing protein | Protein of unknown function DUF45 | SprT homologues |

Table S6. CFU counts of HPUFs and relative CFU of HPUFs to the CFU of non-toxic control genes in that batch of electroporations.

| Assay | Gene | Transformation eficiency (CFU/ng) | Relative CFU |
|---|---|---|---|
| 1 | g073 | 7518,66 | 1,501 |
| | g083 | 6841,98 | 1,366 |
| | g081 | 3705,96 | 0,740 |
| | g074 | 2896,41 | 0,578 |
| | g076 | 2525,25 | 0,504 |
| | g121 | 5010,07 | 1,000 |
| 2 | g084 | 11437,68 | 4,752 |
| | g086 | 8582,75 | 3,566 |
| | g087 | 5257,05 | 2,184 |
| | g089 | 3828,97 | 1,591 |
| | g085 | 3742,34 | 1,555 |
| | g121 | 2407,12 | 1,000 |
| 3 | g090 | 8220,83 | 2,583 |
| | g091 | 8173,69 | 2,568 |
| | g121 | 3182,29 | 1,000 |
| 4 | g135 | 2782,33 | 0,545 |
| | g138 | 4376,30 | 0,858 |
| | g178 | 5101,29 | 1,000 |
| 5 | g181 | 4108,41 | 2,738 |
| | g199 | 3378,13 | 2,251 |
| | g141 | 1924,77 | 1,283 |
| | g167 | 1840,05 | 1,226 |
| | g177 | 1274,59 | 0,849 |
| | g178 | 1500,70 | 1,000 |
| 6 | g209 | 4001,06 | 1,833 |
| | g208 | 3716,65 | 1,703 |
| | g205 | 2074,28 | 0,950 |
| | g204 | 1981,72 | 0,908 |
| | g206 | 1897,88 | 0,870 |
| | g178 | 2182,34 | 1,000 |

| | | | |
|---|---|---|---|
| 7 | g221 | 7703,72 | 2,863 |
| | g219 | 6983,61 | 2,595 |
| | g214 | 5973,63 | 2,220 |
| | g217 | 3265,12 | 1,213 |
| | g210 | 3025,35 | 1,124 |
| | g218 | 2912,75 | 1,082 |
| | g246 | 2691,12 | 1,000 |
| 8 | g224 | 8934,64 | 1,278 |
| | g241 | 4330,18 | 0,619 |
| | g222 | 3826,52 | 0,547 |
| | g236 | 2807,18 | 0,401 |
| | g240 | 1231,91 | 0,176 |
| | g232 | 5,04 | 0,001 |
| | g246 | 6993,21 | 1,000 |
| 9 | g258 | 9596,13 | 5,165 |
| | g259 | 5674,18 | 3,054 |
| | g255 | 3507,08 | 1,888 |
| | g261 | 1693,78 | 0,912 |
| | g256 | 1612,45 | 0,868 |
| | g246 | 1857,86 | 1,000 |
| 10 | g202 | 5655,88 | 0,779 |
| | g111 | 3136,76 | 0,432 |
| | g246 | 7258,62 | 1,000 |
| 11 | g104 | 7950,78 | 1,259 |
| | g098 | 4998,45 | 0,792 |
| | g095 | 3248,04 | 0,514 |
| | g012 | 5753,53 | 0,911 |
| | g150 | 6313,72 | 1,000 |
| 12 | g257 | 11115,59 | 1,458 |
| | g019 | 8301,81 | 1,089 |
| | g150 | 7626,28 | 1,000 |
| 13 | g017 | 7927,71 | 2,188 |
| | g022 | 7196,60 | 1,986 |
| | g023 | 6796,79 | 1,876 |
| | g028 | 5815,51 | 1,605 |
| | g030 | 4788,25 | 1,322 |
| | g121 | 3622,92 | 1,000 |
| 14 | g094 | 3898,16 | 0,729 |
| | g043 | 4278,43 | 0,801 |
| | g137 | 2354,55 | 0,441 |
| | g121 | 5344,62 | 1,000 |
| 15 | g093 | 2255,86 | 0,518 |
| | g105 | 3443,40 | 0,790 |

| | | | |
|---|---|---|---|
| | g124 | 4826,75 | 1,108 |
| | g121 | 4357,29 | 1,000 |
| 16 | g033 | 350,31 | 0,314 |
| | g044 | 463,52 | 0,415 |
| | g045 | 709,08 | 0,635 |
| | g049 | 448,72 | 0,402 |
| | g061 | 1006,06 | 0,902 |
| | g064 | 195,54 | 0,175 |
| | g065 | 2587,00 | 2,318 |
| | g178 | 1115,91 | 1,000 |
| 17 | g067 | 1436,74 | 1,428 |
| | g069 | 2113,21 | 2,101 |
| | g070 | 450,43 | 0,448 |
| | g071 | 1711,08 | 1,701 |
| | g002 | 1298,55 | 1,291 |
| | g004 | 2168,49 | 2,156 |
| | g011 | 3028,42 | 3,010 |
| | g178 | 1005,96 | 1,000 |
| 18 | g014 | 4094,60 | 1,432 |
| | g016 | 3238,41 | 1,133 |
| | g092 | 196,48 | 0,069 |
| | g096 | 1367,99 | 0,479 |
| | g099 | 1828,75 | 0,640 |
| | g100 | 316,63 | 0,111 |
| | g101 | 2402,88 | 0,841 |
| | g178 | 2858,48 | 1,000 |
| 19 | g106 | 718,45 | 0,684 |
| | g112 | 1076,88 | 1,026 |
| | g117 | 1118,44 | 1,065 |
| | g126 | 987,45 | 0,940 |
| | g127 | 726,34 | 0,692 |
| | g178 | 1049,94 | 1,000 |
| 20 | g132 | 1196,28 | 0,607 |
| | g130 | 1344,71 | 0,682 |
| | g129 | 2372,85 | 1,204 |
| | g178 | 1970,70 | 1,000 |
| 21 | g052 | 2041,84 | 2,041 |
| | g029 | 1024,44 | 1,024 |
| | g102 | 2471,80 | 2,471 |
| | g128 | 1079,21 | 1,079 |
| | g178 | 1000,47 | 1,000 |
| 22 | g136 | 3658,43 | 0,396 |
| | g178 | 9248,83 | 1,000 |

Table S7. CFU values for ϕR1-RT encoded holins and endolysin relative to CFU of **control non-toxic gene g119.**

| Assay | Gene | Length (bp) | Predicted function | Transformation eficiency (CFU/ng) | Relative CFU |
|---|---|---|---|---|---|
| 1 | g122 | 489 | lysin | 2053,97 | 1,215 |
| | g252 | 162 | holin | 2267,63 | 1,342 |
| | g253 | 657 | holin | 1658,58 | 0,981 |
| | g119 | 591 | structural protein | 1690,36 | 1,000 |

Figure S1. Map of the plasmid construct pU11L4 with KpnI-PstI linker and luxAB. All the control genes and the HPUF genes were cloned in the linker region.

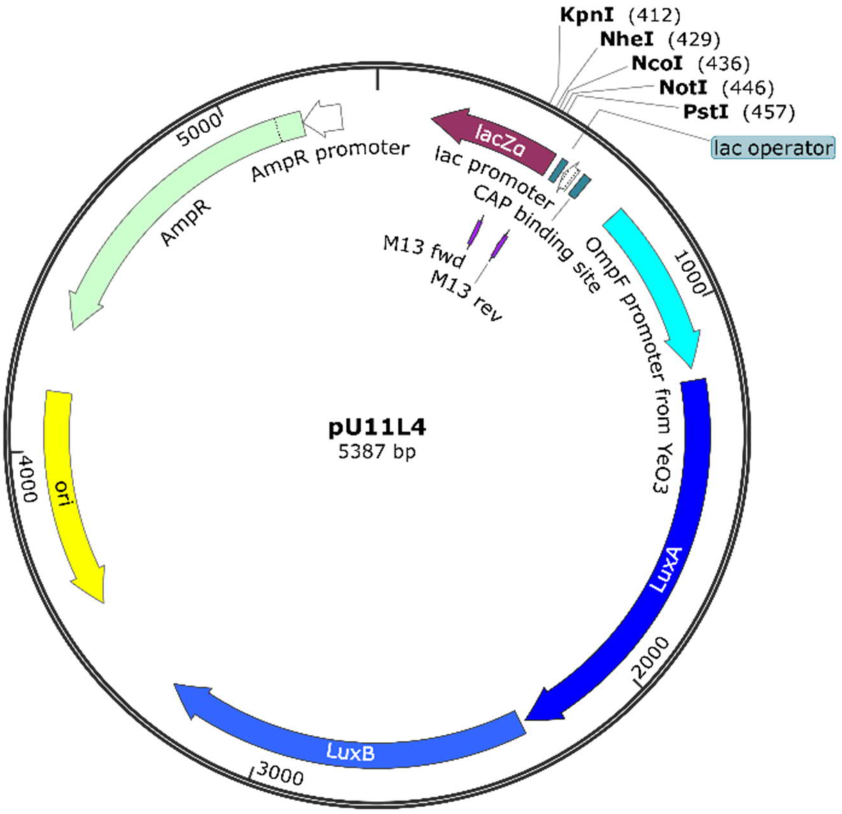

Figure S2. Inter-assay variability of the screening assay. In order to calculate the inter-assay variability of the relative CFU values of toxic hits with respect to the control gene g178, two separate ligations of the genes to the pU11l4 was carried out and each ligation mix was electroporated and plated in replicates of three.

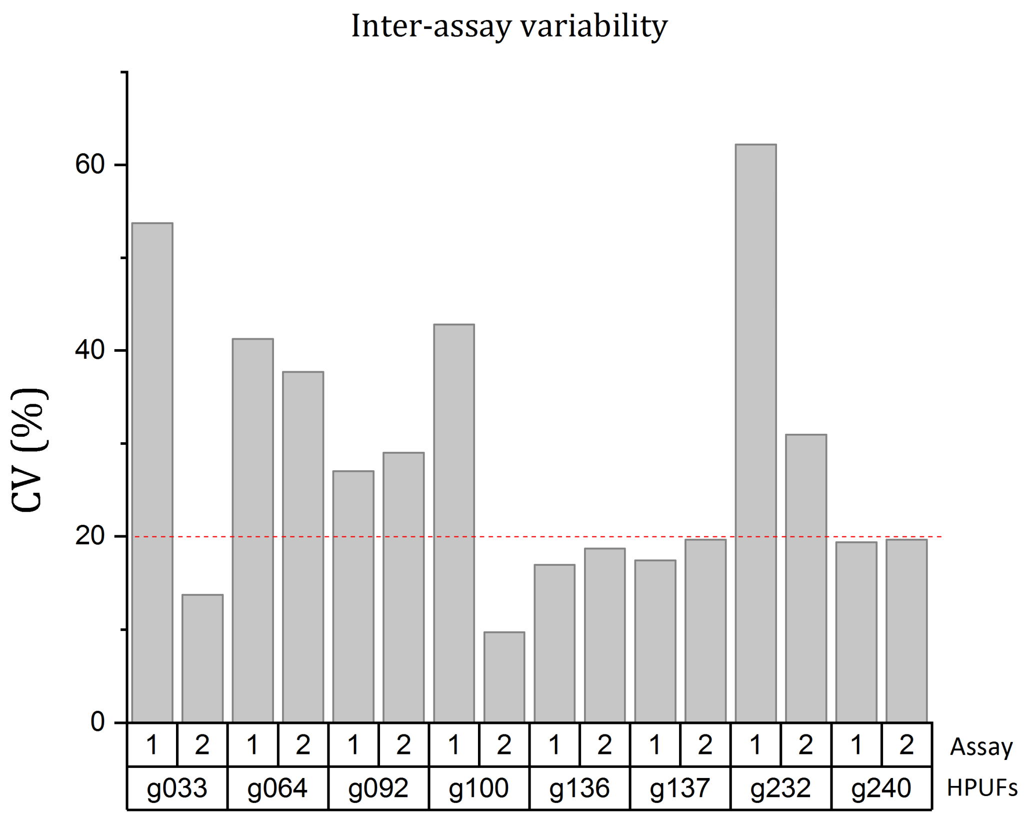

Supplement: Supplementary file 1 [file viruses-11-01057-s001.pdf]
